# A comparison of medetomidine and its active enantiomer dexmedetomidine when administered with ketamine in mice

**DOI:** 10.1186/1746-6148-9-48

**Published:** 2013-03-13

**Authors:** Wesley M Burnside, Paul A Flecknell, Angus I Cameron, Aurélie A Thomas

**Affiliations:** 1 Comparative Biology Centre, Newcastle University, Framlington Place, Newcastle upon Tyne, NE2 4HH, United Kingdom; 2 School of Veterinary Medicine, College of Medical, Veterinary and Life Sciences, University of Glasgow, Bearsden Road, Glasgow, G61 1QH, United Kingdom; 3 Boyd Orr Centre for population and ecosystem health, Institute of Biodiversity, Animal Health and Comparative Medicine, College of Medical, Veterinary and Life Sciences, University of Glasgow, Glasgow, G12 8QQ, United Kingdom

**Keywords:** Alpha-2 agonists, Dexmedetomidine, Drug administration route, General anaesthesia, Hypoxaemia, Ketamine, Mouse, Medetomidine, Subcutaneous injection, Supplemental oxygen

## Abstract

**Background:**

Medetomidine-ketamine (MK) and dexmedetomidine-ketamine (DK) are widely used to provide general anaesthesia in laboratory animals, but have not been compared directly in many of these species, including rodents. This study aimed to compare the onset and depth of anaesthesia, and changes in vital signs, after intraperitoneal (IP) or subcutaneous (SC) administration of ketamine (75 mg kg^-1^) combined with medetomidine (1 mg kg^-1^) or dexmedetomidine (0.5 mg kg^-1^) using a randomised semi-crossover design with ≥ 48 hours between treatments in 10 male and 10 female mice. Each mouse was anaesthetised twice using the same administration route (IP or SC): once with each drug-ketamine combination. Anaesthetised mice were monitored on a heating pad without supplemental oxygen for 89 minutes; atipamezole was administered for reversal. The times that the righting reflex was lost post-injection and returned post-reversal were analysed using general linear models. Tail-pinch and pedal reflexes were examined using binomial generalized linear models. Pulse rate (PR), respiratory rate (*f*r), and arterial haemoglobin saturation (S_p_O_2_) were compared using generalized additive mixed models.

**Results:**

There were no significant differences among treatments for the times taken for loss and return of the righting reflex, or response of the tail-pinch reflex. The pedal withdrawal reflex was abolished more frequently with MK than DK over time (*P* = 0.021). The response of PR and S_p_O_2_ were similar among treatments, but *f*r was significantly higher with MK than DK (*P* ≤ 0.0005). Markedly low S_p_O_2_ concentrations occurred within 5 minutes post-injection (83.8 ± 6.7%) in all treatment groups and were most severe after 89 minutes lapsed (66.7 ± 7.5%). No statistical differences were detected in regards to administration route (*P* ≤ 0.94).

**Conclusions:**

This study failed to demonstrate clinical advantages of the enantiomer dexmedetomidine over medetomidine when combined with ketamine to produce general anaesthesia in mice. At the doses administered, deep surgical anaesthesia was not consistently produced with either combination; therefore, anaesthetic depth must be assessed before performing surgical procedures. Supplemental oxygen should always be provided during anaesthesia to prevent hypoxaemia.

## Background

Injectable anaesthesia is often considered the method of choice for experimental procedures in small rodents [1]. Alpha-_2_ agonists are routinely used in combination with other drugs to produce anaesthesia. One benefit of α_2_ agonists is that their effects can be reversed using a specific antagonist, atipamezole [2]. Of these agents, medetomidine is a popular choice in various species due to its high α_2_ receptor selectivity compared to romifidine, detomidine, and xylazine. Medetomidine, a racemate of the stereoisomers levomedetomidine and dexmedetomidine, is a sedative and analgesic that acts centrally on α_2_ adrenergic receptors largely concentrated in the locus coeruleus of the brain stem to decrease sympathetic tone. Peripherally, medetomidine activates postsynaptic α_2_ adrenergic receptors in vascular smooth muscle to cause vasoconstriction. Its main side effects include bradycardia, hypotension, respiratory depression, hypothermia and diuresis [3-7]. Hypotension is a result of centrally mediated effects on sympathetic tone that may be apparent after the initial peripheral vasoconstrictive effects pass [2,8-10]. This phenomenon is also observed during the administration of other α_2_ agonists [11,12].

Dexmedetomidine has been identified as the active enantiomer of medetomidine, yet the effects of levomedetomidine have been debated. Unlike medetomidine and dexmedetomidine, levomedetomidine alone does not appear to have effects on the cardiovascular system and only provides sedation or analgesia at high doses in rats and mice [3]. Its clearance is four times more rapid in the dog [13]. A second study in the dog compared a high dose and low dose of levomedetomidine by administration of an initial intravenous (IV) bolus (10 or 80 μg kg^-1^) followed by two-hour constant rate infusions of levomedetomidine alone (25 or 200 μg kg^-1^ h^-1^) with or without a single dose dexmedetomidine (10 μg kg^-1^); the study concluded that the combined administration of a high dose of levomedetomidine and dexmedetomidine significantly reduced sedation scores and increased the incidence of bradycardia [14]. In human cell culture, levomedetomidine was described as an “inverse” agonist because it uncoupled active α_2_ adrenergic receptors by decreasing [Ca^2+^] and inhibiting cAMP function [15]. It has been suggested that the sedative effects of dexmedetomidine alone may therefore be more predictable and the cardiovascular side effects should be minimised compared to the racemic mixture. However, no significant difference was observed when levomedetomidine was co-administered with dexmedetomidine until levomedetomidine exceeded eight times the normal racemic preparation [13]. Although some studies have suggested that dexmedetomidine is superior to medetomidine [13,16], this may not be the case.

A handful of studies have compared the sedative effects of medetomidine and dexmedetomidine in the mouse and rat [3], dog [13,17,18], cat [16,19,20], and sheep [10]. All of these studies except two concluded there were no clinical advantages of dexmedetomidine over medetomidine [13,16]. Although there were no significant differences, one study concluded that “dexmedetomidine tended to be slightly more potent than the equivalent dose of medetomidine” and may provide more predictable sedation and analgesia in the dog [13]. A more recent comparison reported significantly greater muscle relaxation in medetomidine-treated cats [16].

Ketamine in combination with medetomidine (MK) has been widely used as an anaesthetic for laboratory rats and mice [1,7,21-24]. Ketamine is a centrally acting NMDA-receptor antagonist that rapidly induces dissociative anaesthesia while providing analgesia. Its side effects include tachycardia, dysphoria, and muscle rigidity. When combined with an α_2_ agonist, general anaesthesia may be attained. The muscle relaxant properties of the α_2_ agonist offset the rigidity induced by ketamine and co-administration decreases the effective dose of each drug [5-7].

The introduction of dexmedetomidine as a veterinary product has been accompanied by the withdrawal of medetomidine in some countries (e.g. the USA). Consequently, use of dexmedetomidine in combination with ketamine (DK) has increased, with dose rates based on assumptions of potency derived from the relatively few studies in other species. Because dexmedetomidine was identified as the active enantiomer of medetomidine, the dose of dexmedetomidine administered is normally half that of the medetomidine; this is due to the absence of levomedetomidine that comprises 50% of the medetomidine racemate. Three studies in other species administered equal amounts of ketamine combined with doses of medetomidine or dexmedetomidine assumed by the researchers to be equipotent. A study of the golden-headed lion tamarin *Leontopithecus chrysomelas* concluded that DK provided greater anaesthetic depth because the length of time it took tamarins treated with DK to walk after standing was significantly greater [25]. Similarly, a field experiment in the Chinese water deer *Hydropotes inermis* also observed a more rapid recovery with MK, as well as significantly faster immobilisation [26]. There were no significant differences between MK and DK when administered to the Bennett’s wallaby *Macropus rufogriseus*[27]. Although these studies support interspecies variation in response to MK and DK administration, currently there appear to be no data directly comparing MK and DK in laboratory rodents.

This study aimed to compare the onset and depth of anaesthesia, and changes in vital signs, after intraperitoneal (IP) or subcutaneous (SC) administration of MK or DK combinations in mice.

## Methods

### Animals

Twenty C57BL/6N mice, 10 male and 10 female were acquired from a commercial breeder (Charles River UK Ltd., Kent, UK). These mice were acclimated two weeks prior to the study in an animal room maintained at 22 ± 1°C and 35% humidity, on a 12-hour light-dark cycle (beginning at 07:00) with 15 to 20 air changes hour^-1^; regular serological monitoring ensured this facility is free from all recognised respiratory pathogens of rodents. A positive-pressure individually ventilated cage system (Maxiseal, Arrowmight, Hereford, UK) was used to house a maximum of six same-sex mice cage^-1^. Each cage contained aspen woodchip bedding (BS&S Ltd., Edinburgh, UK) and nesting material (shredded paper, DBM Food Hygiene Supplies Ltd., Broxburn, UK). Food (CRM (*P*), Special Diets Services, Essex, UK) and tap water were provided *ad libitum*. At the time of the study, the mice were 6 weeks old and ear notched for identification; males weighed 23 ± 1.4 g and females weighed 19 ± 0.7 g.

### Experimental design

Four anaesthetic treatments were evaluated by a randomised semi-crossover design with ≥ 48 hours between treatments. Ketamine (75 mg kg^-1^, Ketalar™ Injection, Pfizer Ltd., Sandwich, UK) was combined with medetomidine (1 mg kg^-1^, Domitor®, Janssen Animal Health, Basingstoke, UK) or dexmedetomidine (0.5 mg kg^-1^, Dexdomitor®, Janssen Animal Health). Each mouse was randomly anaesthetised with one drug-ketamine combination (e.g. medetomidine) administered by the IP or SC route; after a minimum of 48 hours, each mouse was anaesthetised again with the other drug-ketamine combination (e.g. dexmedetomidine) administered by the same route as its first treatment. Data were collected from 06 to 17 June 2011 between 9:00 and 13:00 daily to ensure time of day did not affect results. No more than three mice were concurrently anesthetised to allow enough time to monitor each individual; the injection of each mouse was staggered by 5 minutes. The same operator collected all the data obtained in this study.

This study was carried out in accordance with project and personal licenses granted under the United Kingdom’s Animals (Scientific Procedures) Act (1986) and the Newcastle University Ethical Review Committee specifically approved this study (PPL 60/4126).

### Procedure

The time from injection to loss of the righting reflex (LORR) was recorded when the mouse appeared immobile and was physically rolled into lateral recumbency by hand to verify that the righting reflex was abolished. Each mouse was placed on a digital thermoregulated heating pad (37°C, 507220F, Harvard Apparatus, Kent, UK) to breathe room air; protective eye lubricant was applied (Puralube®, Pharmaderm, Melville, NY, USA). The hair of the right thigh was clipped and a pulse oximeter (MouseOx®, Starr Life Sciences Corp., Oakmont, PA, USA) probe was placed to monitor pulse rate (PR) and arterial haemoglobin saturation (S_p_O_2_). In addition to PR and S_p_O_2_, the tail-pinch and pedal withdrawal reflexes (presence or absence determined “by hand”), and respiration rate (*f*r; determined by observation of chest wall movement) were monitored for 89 minutes from 5 minutes post-injection at 7-minute intervals. The PR and S_p_O_2_ were averaged from values collected every 0.2 seconds (MouseOx®) over a 30-second time interval for each sample time point. Regardless of any parameters, the effects of medetomidine or dexmedetomidine were antagonised at 89 minutes post-injection by atipamezole (5 mg kg^-1^, Antisedan®, Janssen Animal Health) administered by the same route as induction. The time until return of the righting reflex (RORR) was recorded as the time from atipamezole administration until the mouse rolled unassisted from lateral to ventral (sternal) recumbency. Then, mice were placed in a fan-assisted incubator (25°C, MediHeat™, Peco Servies Ltd., Cumbria, UK) until full recovery.

### Statistical analysis

General linear models (glms) were used to analyse the times until LORR and RORR. The full models included drug, administration route, bodyweight, and sex as explanatory variables with drug and administration route included as an interaction term. The optimal model was determined by the stepwise removal of variables to reach the lowest Akaike’s information criterion (AIC). A reduction of ≥ 5 in AIC was considered indicative of a significant improvement in model fit.

The presence of tail-pinch and pedal withdrawal reflexes was evaluated with generalized linear models (GLMs). Models included a binomial error structure to account for the binary nature of the response variable. Explanatory variables in the full model included drug, administration route, and time each measurement was taken, with drug and administration route included as an interaction term. Stepwise removal of variables was used to determine the optimal model. Model residuals were examined with an auto-correlation function (acf) and showed that the pedal withdrawal reflex models were temporally auto-correlated. Pedal withdrawal reflex was better explained with a binomial generalized additive model with an auto-regressive correlation structure to account for the temporal dependence between residuals. Stepwise removal of variables was used to determine the optimal model.

The PR, *f*r, and S_p_O_2_ were examined with generalized additive mixed effects models (GAMMs) to allow for potential non-linear relationships among variables as well as violations of independence within the data. The full models for each response variable included the other two response variables as additional explanatory variables for each model (e.g. the model for PR included *f*r, and S_p_O_2_). Drug, administration route, time, bodyweight, and sex were also included as explanatory variables for each of the three response variables. Where an explanatory variable appeared to demonstrate non-linear relationships with the response (based on primary visual assessment of data), they were included as smooth terms in the full model (Additional file 1). *Mouse* was included as a normally distributed random effect to account for pseudo-replication in the semi-crossover design. Optimal models were reached by the stepwise removal of variables, and replacement of non-significant smooth terms with linear ones. Final models were tested with acfs for the presence of temporal auto-correlation. Optimal correlation structures were selected by AIC to correct for this auto-correlation. All models had this best accounted for by auto-regressive moving-average correlation structures. These were then applied to the full model and stepwise removal of explanatory variables was performed again.

All analyses were performed using R version 2.14.0 (R Foundation for Statistical Computing; http://www.R-project.org). A significance level of *P* ≤ 0.05 was used during interpretation of these analyses and all means are reported as the mean ± standard deviation in the text.

## Results

**Figure 1:**
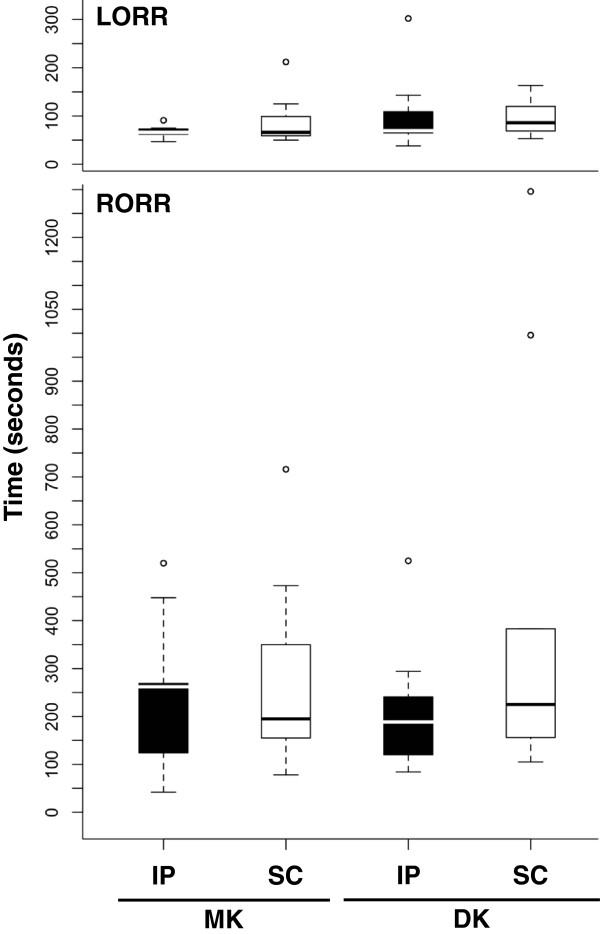
**Loss and return of the righting reflex.** Mean time (seconds) until loss of the righting reflex (LORR) and return of the righting reflex (RORR) after administration of medetomidine-ketamine (MK) or dexmedetomidine-ketamine (DK) by the intraperitoneal (IP) or subcutaneous (SC) route. The time to LORR was not significantly affected by drug (*P* = 0.29) or administration route (*P* = 0.71). Similarly, the time until RORR was not significantly affected by drug (*P* = 0.61) or administration route (*P* = 0.18).

The time until LORR was not significantly affected by drug (glm, *F* = 1.14, *P* = 0.29), administration route (glm, *F* = 0.14, *P* = 0.71; Figure 1), or individual bodyweight (glm, *F* = 3.30, *P* = 0.078). Similarly, the time until RORR was not significantly affected by drug (glm, *F* = 0.26, *P* = 0.61), administration route (glm, *F* = 1.86, *P* = 0.18; Figure 1), or individual bodyweight (glm, *F* = 0.16, *P* = 0.69).

**Figure 2:**
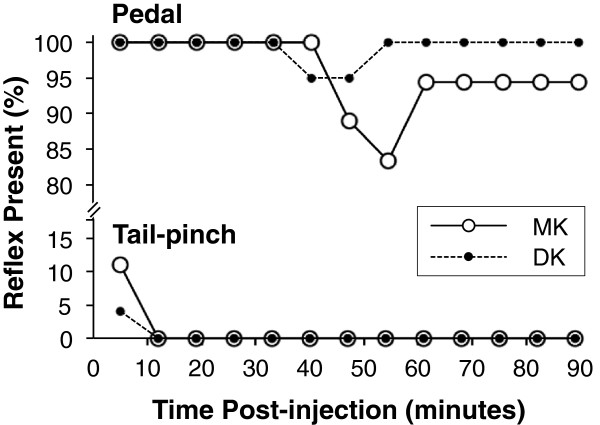
**Tail-pinch and pedal reflexes.** Percentage of individuals with present tail-pinch and pedal withdrawal reflexes after administration of medetomidine-ketamine (MK) or dexmedetomidine-ketamine (DK) over time. Loss of the tail-pinch reflex did not significantly differ between drug combination (*P* = 0.36). Pedal withdrawal reflex loss was not consistently achieved by either drug combination, but was more frequent with MK than DK over time (*P* = 0.021).

Loss of the tail-pinch reflex occurred by 12 minutes post-injection and did not significantly differ between MK and DK (GLM, *z* = 0.92, *P* = 0.36; Figure 2). Neither drug consistently achieved loss of the pedal withdrawal reflex by either administration route, but was more frequent with MK than DK over time (GLM, *z* = 2.3, *P* = 0.021; Figure 2).

**Figure 3:**
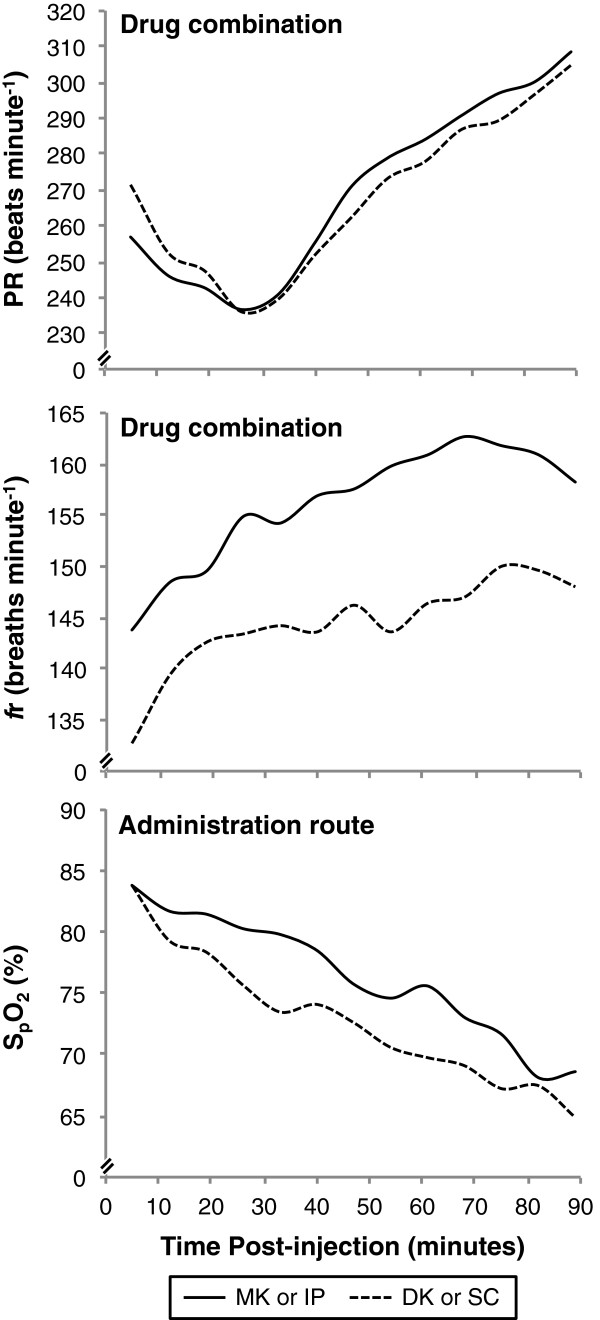
**Vital signs.** Mean pulse rate (PR) and respiratory rate (*f*r) after administration of medetomidine-ketamine (MK) or dexmedetomidine-ketamine (DK), and arterial haemoglobin saturation (S_p_O_2_) after drug administration by the intraperitoneal (IP) or subcutaneous (SC) route over time. Although means were not used to determine statistical significance, they effectively display data trends.

Pulse rate was not significantly different between drug (GAMM, *t* = 0.48, P = 0.63; Figure 3) or administration route (GAMM, *t* = 0.47, *P* = 0.64), bodyweight (GAMM, *t* = 0.008, P = 0.99) or sex (GAMM, t= 1.26, *P* = 0.21). It was negatively correlated with time until about 47 minutes post-injection, and positively correlated thereafter (GAMM, *F* =16.59, e.d.f. = 6.13, *P* ≤ 0.0005). Pulse rate was also positively correlated with S_p_O_2_ until about 77% S_p_O_2_, after which the correlation became negative (GAMM, *F* = 9.20, e.d.f. = 2.74, *P* ≤ 0.0005).

The *f*r was significantly higher with MK than DK (GAMM, *t* = 4.047, *P* ≤ 0.0005; Figure 3). Administration route did not significantly affect *f*r (GAMM, *t* = 0.077, *P* = 0.94). The correlation between *f*r and S_p_O_2_ was negative until approximately 76% S_p_O_2_, at which point the correlation becomes positive (GAMM, *F* = 19.19, e.d.f. = 1.86, *P* ≤ 0.0005).

The S_p_O_2_ was not affected by drug (GAMM, *t* = 0.76, *P* = 0.45) or administration route (GAMM, *t* = 1.75, *P* = 0.081; Figure 3), but positively correlated with bodyweight (GAMM, *t* = 2.85, *P* = 0.0046) and was significantly lower in males than females (GAMM, *t* = 2.88, *P* = 0.0041). The S_p_O_2_ was negatively correlated with PR (GAMM, *t* = 6.87, *P* = 0.0001) and *f*r (GAMM, *t* = 4.240, *P* ≤ 0.0005). It was positively correlated with time until about 47 minutes post-injection and negatively correlated thereafter (GAMM, *F* = 50.87, e.d.f. = 2.86, *P* ≤ 0.0005).

## Discussion

Medetomidine and dexmedetomidine are routinely administered alone or in combination with other drugs including ketamine; however, to the best of our knowledge, only a handful of peer-reviewed studies have aimed to compare the sedative and analgesic properties of these drugs alone and even fewer compared their use in conjunction with ketamine.

Our study found no significant difference in LORR and RORR times based on drug, administration route, or individual bodyweight. Loss of the tail-pinch reflex occurred by 12 minutes post-injection in all treatments and did not significantly differ between MK and DK. Neither drug consistently achieved loss of the pedal withdrawal reflex by either administration route, but loss was more frequent with MK than DK over time. Pulse rate was not significantly different among treatments, but *f*r was significantly higher with MK than DK; administration route did not significantly affect *f*r. The S_p_O_2_ was not affected by drug or administration route, but positively correlated with bodyweight and was significantly lower in males than females. On average, the S_p_O_2_ of the mice was markedly low (<85%) within 5 minutes post-injection and decreased severely (to < 70%) by 89 minutes.

Many studies that directly compared medetomidine and dexmedetomidine as a sedative or premedication found no clinically significant differences in various species [3,15-19]. In a collection of experiments in laboratory rodents, IV sedation with medetomidine and its enantiomers were found to induce similar levels of hypotension, bradycardia and loss of the mydriatic response; levomedetomidine had no cardiovascular or pupillary effect when administered IV [3]. In terms of analgesia, the same group found that all three drugs (0.01 to 1 mg kg^-1^ medetomidine; 0.01 to 0.1 mg kg^-1^ dexmedetomidine; 0.3 to 10 mg kg^-1^ levomedetomidine) appeared to inhibit the acetic acid-induced writhing response in mice. Ultimately, these studies concluded there were no major differences between medetomidine and dexmedetomidine [3]. Based on the results of the writhing test, levomedetomidine may have analgesic properties. This lack of differences reported in these previous studies when medetomidine and dexmedetomidine were administered alone supports our current findings that there was no apparent clinical difference between these drugs administered in combination with ketamine.

The drug dosages used in this study represent commonly reported MK combinations administered to mice [1,7,22-24]. The dexmedetomidine dose (0.5 mg kg^-1^) used was calculated as half the medetomidine dose (1 mg kg^-1^) because it lacks the levomedetomidine component present in the racemic mixture. We therefore expected the doses of medetomidine and dexmedetomidine to be equipotent.

The recommended dose of atipamezole to reverse the effects of these drugs is 5 mg kg^-1^ for 1 mg kg^-1^ of medetomidine or 0.5 mg kg^-1^ of dexmedetomidine [22], but its reported quality of reversal is inconsistent [21]. We used the time until RORR as a measure of MK and DK reversibility by atipamezole; there were no significant differences, but there was a wide variation in recovery times (293 ± 258 seconds).

Depending on the procedure to be performed, the ideal anaesthetic depth may vary. In practice, depth of anaesthesia is generally defined by the loss of specific reflexes as the depth of anaesthesia progresses from sedation to surgical anaesthesia. Sedation is the first level; in mice, locomotion ceases, respiration slows, and the head and tail are relaxed. Next, light anaesthesia occurs when the righting reflex is lost, but the mouse will respond to painful stimuli. Surgical depth of anaesthesia is achieved when the tail-pinch and pedal withdrawal reflexes are lost.

Subcutaneous administration of 0.3 mg kg^-1^ of dexmedetomidine abolished the righting reflex in rats, but paradoxical excitability was observed at the 1 mg kg^-1^ dose; neither medetomidine nor dexmedetomidine induced sustainable loss of the pedal withdrawal reflex indicating light anesthesia [3]. Similarly, the doses of MK and DK administered in our study induced LORR (89 ± 51 seconds), but, based on their failure to abolish the pedal withdrawal reflex throughout the time post-injection, may not provide appropriate anaesthetic depth suitable for surgical procedures (Figure 2). These findings were consistent with other reports of MK administration in mice [1,22]. Studies that compared depth of anaesthesia between MK and DK suggested that DK produced a greater anaesthetic depth [25,26]; conversely, our study observed significantly deeper anaesthesia from MK (Figure 2). These inconsistent findings could be due to species differences among mice, golden-headed lion tamarins and Chinese water deer. Similarly, differences among different strains of the same species (e.g. a BALB/c or C57BL/6N background in mice) have been reported in the field of analgesia and anaesthesia [28-31].

Normal resting PR in the mouse ranges from 350 to 600 beats minute^-1^[32]. Bradycardia was apparent for approximately 26 minutes post-injection when the average PR reached a minimum (236 ± 35 beats minute^-1^); the PR steadily increased thereafter (Figure 3). This dramatic decrease in PR was attributed to reflex bradycardia, a baroreceptor response that compensates for initial α_2_-induced peripheral vasoconstriction and hypertension. Other studies also found no differences in PR when MK and DK were compared [26,27]. These well-understood physiological mechanisms support that the PR responded as expected when an α_2_ agonist is administered, while these studies support that there are no clinical differences between MK or DK administered IV or SC in mice.

The normal resting *f*r in the mouse ranges from 80 to 200 breaths minute^-1^[32]. The mice treated with DK (144 ± 17 breaths minute^-1^) had significantly lower *f*r than those treated with MK (156 ± 15 breaths minute^-1^; Figure 3), but the difference does not appear to be clinically significant based on the normal resting respiratory rate and the lack of difference among treatments with regards to S_p_O_2_. The increase in *f*r over time could be a combined response to a rapid decrease in arterial partial pressure of oxygen (P_a_O_2_) and potential increase in arterial partial pressure of carbon dioxide (P_a_CO_2_). Although P_a_CO_2_ is the primary trigger stimulating the respiratory drive, abnormally low P_a_O_2_ (≤ 60 mmHg) alone can have a role as well, referred to as the “hypoxic ventilatory response” [33,34]. Direct measurements of P_a_O_2_ and P_a_CO_2_ would be necessary to confirm this. On observation, respiratory patterns of individual mice appeared shallower over time; this may account for the steady increase in *f*r, while the S_p_O_2_ continued to decrease. Respiratory depression, also known as hypoventilation, caused by α_2_ agonists was suspected in this study because depression of the central nervous system following the activation of α_2_ agonist receptors includes depression of respiratory centres. [4,6,35]. Measurements of tidal volume and end-tidal carbon dioxide of the mice would be necessary to confirm this. The effect of MK and DK on *f*r in other studies was inconsistent [25-27]. This lack of consistency could be also attributed to variation among species, or may require further examination to verify the physiological process that occurred in our study.

Although S_p_O_2_ was not significantly different among treatment groups, there was an interesting relationship between sex and bodyweight. Although a significant positive correlation existed between S_p_O_2_ and bodyweight, male mice had a significantly lower S_p_O_2_ than females. An autocorrelation exists between sex and weight: under normal circumstances in mice of a given age, males have a predictably greater bodyweight than females; however, an unknown component of sex sets the average S_p_O_2_ of male mice lower than females. A study in rats suggested that females may have a more efficient oxygen transport system than males related to a higher pulmonary compliance [36], while another found that virgin female rats at the onset of sexual maturity have a greater gas-exchange surface area than male rats [37]. In our study, a greater pulmonary compliance of prepubescent female mice could contribute to the significant differences observed between sexes. A specific study would be required to further explain the relationship we observed in mice and determine if males and females have different requirements for oxygen under anaesthesia.

Hypoxaemia is a significant cause of mortality in anesthetised mice, yet supplemental oxygen is not commonly used [7]. In order to mimic common laboratory practice, supplemental oxygen was not supplied in this study. The S_p_O_2_ was markedly low within 5 minutes post-injection (83.8 ± 6.7%) and decreased severely to 66.7 ± 7.5% by 89 minutes (Figure 3). Although hypoxaemia was anticipated based on previous studies, S_p_O_2_ levels were lower than expected [25-27]. This large decrease in S_p_O_2_ could be due the monitoring method employed in our study: pulse oximetry.

Pulse oximetry was used in this study because it provides a simple, non-invasive means of monitoring respiratory function; however, pulse oximetry has limitations. Readings become less reliable at lower oxygen saturation levels (< 70 to 75%) [38,39]. This is not usually a significant problem because levels less than 80% require corrective action when supplemental oxygen is not supplied; this threshold increases to 90% for individuals receiving supplemental oxygen. Additionally, peripheral vasoconstriction and hypothermia, side effects of medetomidine, could have prevented accurate readings due to a lack of perfusion in the location of the probe [4-7]. Although we did not monitor core temperature, the heating pad used throughout the experiment should have helped to prevent hypothermia. In a previous study in rats, the pulse oximeter readings were comparable to arterial blood gas values [40]. Other sources of error could include mechanical artifacts from improper probe placement and electromagnetic interference, but are less likely [39].

There were no significant differences between IP and SC administration as the times of onset and anaesthetic depths were similar. This was unexpected because IP administration was anticipated to result in a more rapid onset, greater first pass extraction of the anaesthetic agents by the liver, and possible consequent reduction in efficacy based on previous work [41]. Despite this potential uncertainty as to dose equivalence, SC dosing may be considered preferable to prevent additional stress to the animal, as well as potential damage to internal organs that may occur by IP delivery; it may also be more a more reliable route because IP injections have been associated with a high partial failure rate [42].

The rigorous statistical model selection employed ensured that a dynamic and flexible co-variance structure was appropriately applied to the error structures of time-series models. *Mouse* was included as a random effect to account for the lack of independence that resulted from subjecting each mouse to both drug combinations (the semi-crossover design). Appropriate model error structures accounted for the repeated measures taken sequentially from each mouse. The semi-crossover design allowed us to reduce the number of animals required for the study, reuse mice for multiple treatments, and account for physiological differences among individuals. Because of the lack of independence between time points within these data (as for any analysis of a time-series), displaying mean values for each time-point can only be used to demonstrate overall data trends (Figure 3). More difficult to interpret graphically and beyond the scope of this study, the appropriate plots demonstrating the statistical nature of these relationships required individual plots for each mouse (Additional file 2).

The statistical analyses of related previous work have relied heavily on repeated measures analysis of variance (ANOVA) [13,14,19,25-27,43]. While these tests account for a degree of dependence between data points, the mixed effects modeling approach we utilised is more flexible and robust than more common analyses. Whereas a repeated measures ANOVA can be used to detect linear dependencies between response and explanatory variables, the models used here can be used to explain non-linear autocorrelation (e.g. a decay in dependence as time between data points increases) [44,45]. Modern data analysis techniques could be used to refine experimental protocol and more powerful methodologies could be encouraged in laboratory animal medicine to reduce the numbers of individuals used in studies.

Our study failed to demonstrate clinical differences in the use of MK or DK administered either IP or SC in the mouse. We also demonstrated the need for appropriate oxygen supplementation, even during short or minor procedures. Further research should examine the effects of MK and DK in different mouse strains. Blood pressure monitoring may have demonstrated the relationship between the initial hypertension expected from the peripheral vasoconstriction induced by α_2_ agonists and the initial bradycardia observed. Then, the centrally mediated decrease in blood pressure reported to follow may correlate with the steady increase in PR which occurred after 26 minutes post-injection [2,8-12]. A measure of rectal temperature would ensure the heating pad was appropriately maintaining body temperature.

## Conclusions

Our study failed to demonstrate clinical advantages of the active enantiomer dexmedetomidine over the racemic mixture medetomidine when combined with ketamine for general anaesthesia in mice. These results reassure colleagues working in locations where medetomidine is no longer available that dexmedetomidine can be used with a 50% reduction in dose rate. The doses of MK and DK administered in this study did not consistently produce deep surgical anaesthesia, so anaesthetic depth must be assessed before performing surgical procedures. There was no advantage of the IP administration route compared to the SC injection, but the SC route may be suggested based on previous reports of reduced injury, stress and partial failure rates [42]. Because the S_p_O_2_ levels of all anaesthetised mice were markedly or severely low throughout this study, supplemental oxygen should always be provided regardless of the length of the procedure.

## Abbreviations

acfs: Auto-correlation functions; ANOVA: Analysis of variance; AIC: Akaike’s information criterion; DK: Dexmedetomidine-ketamine; fr: Respiratory rate; GAMMs: Generalized additive mixed effects models; glms: General linear models; GLMs: Generalized linear models; IP: Intraperitoneal; IV: Intravenous; LORR: Loss of the righting reflect; MK: Medetomidine-Ketamine; PaCO2: Arterial partial pressure of carbon dioxide; PaO2: Arterial partial pressure of oxygen; PR: Pulse rate; RORR: Return of the righting reflex; SC: Subcutaneous; SpO2: Arterial haemoglobin saturation

## Competing interests

The authors declare that they have no competing interests.

## Authors’ contributions

AAT and PAF conceived and designed this study. WMB collected, analysed, and arranged the data for statistical analysis. AIC performed the statistical analyses. All authors contributed to data interpretation, as well as the drafting, revising, and final approval of this manuscript.

## Supplementary Material

Additional file 1 **Smoothing curves for non-linear relationships.** Estimated smoothing curves of the non-linear parameters of the effect of time on pulse rate (PR) and arterial haemoglobin saturation (S_p_O_2_), as well as the effect of S_p_O_2_ on PR and respiratory rate (*f*r) determined by a series of generalized additive mixed effects models (GAMMs). Dashed lines represent 95% CI. Significant non-linear relationships occurred for PR (*P* ≤ 0.0001) and S_p_O_2_ (*P* < 0.0001) as a smoothing function of time, as well as PR (*P* ≤ 0.0001) and *f*r (*P* < 0.0001) as a smoothing function of S_p_O_2_. Click here for file

Additional file 2 **Plots of vital signs for individual mice.** Individual plots of pulse rate (PR), respiratory rate (*f*r) and arterial haemoglobin saturation (S_p_O_2_) for each mouse by after administration of medetomidine-ketamine (MK) or dexmedetomidine-ketamine (DK) by the intraperitoneal (IP) or subcutaneous (SC) route over time used for statistical analysis. The individual mouse identification number is located at the bottom left corner of each plot. Click here for file
