# Supplementary figures and images for: A comparison of medetomidine and its active enantiomer dexmedetomidine when administered with ketamine in mice
Source: BMC Vet Res. 2013 Mar 13;9:48. doi: 10.1186/1746-6148-9-48 (PMC3605306; doi:10.1186/1746-6148-9-48)

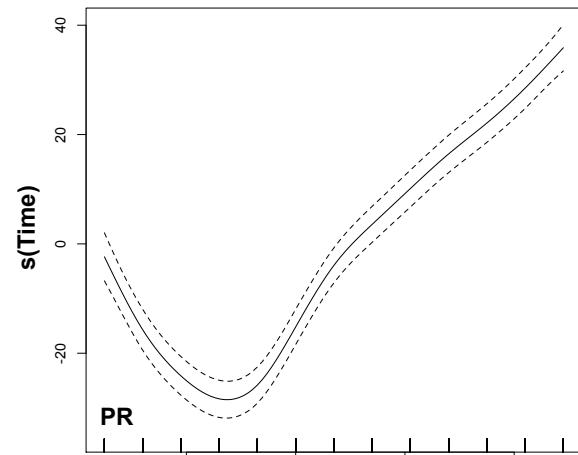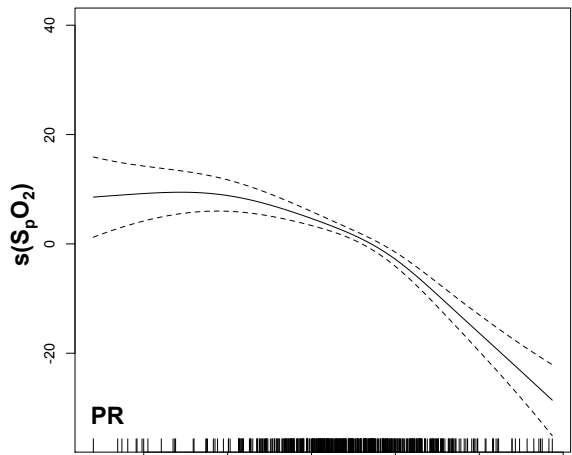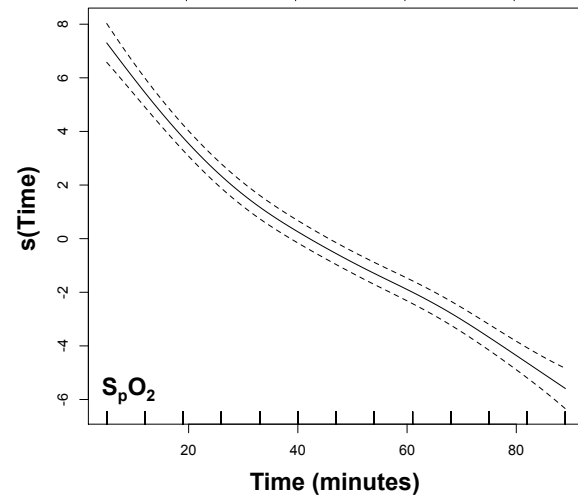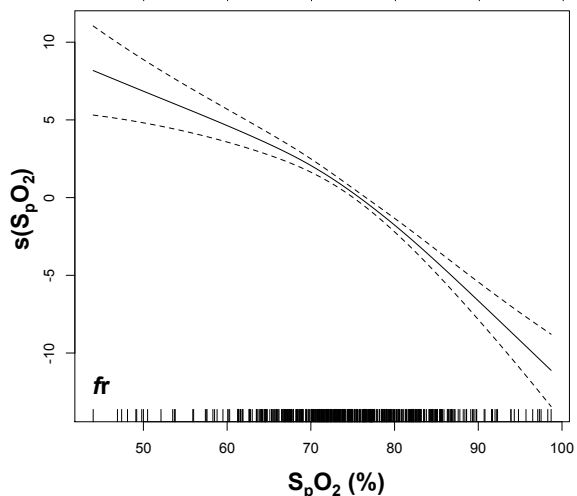

Supplement: Additional file 1 — Smoothing curves for non-linear relationships. Estimated smoothing curves of the non-linear parameters of the effect of time on pulse rate (PR) and arterial haemoglobin saturation (SpO2), as well as the effect of SpO2 on PR and respiratory rate (fr) determined by a series of generalized additive mixed effects models (GAMMs). Dashed lines represent 95% CI. Significant non-linear relationships occurred for PR (P ≤ 0.0001) and SpO2 (P < 0.0001) as a smoothing function of time, as well as PR (P ≤ 0.0001) and fr (P < 0.0001) as a smoothing function of SpO2. [file 1746-6148-9-48-S1.pdf]

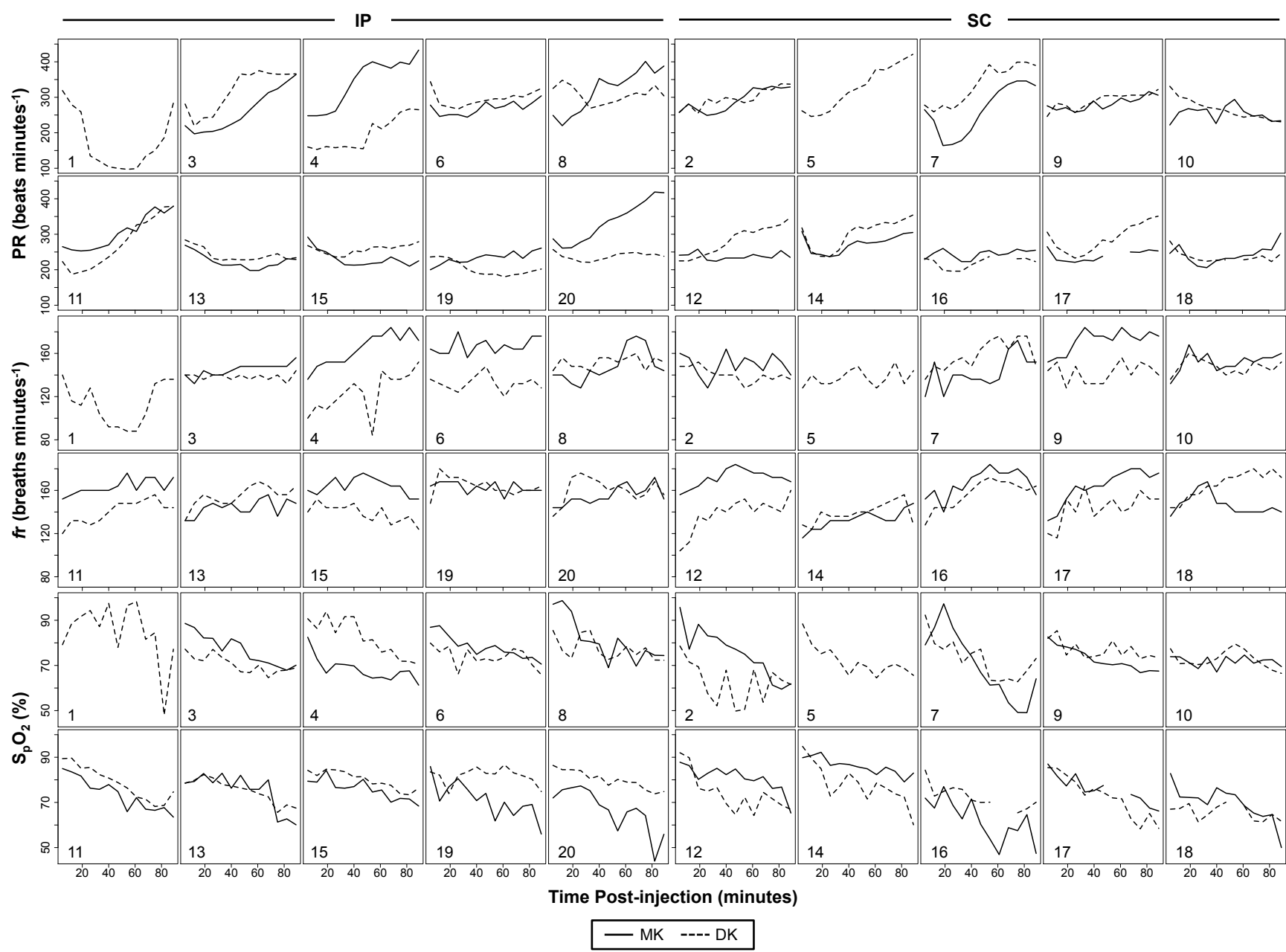

Supplement: Additional file 2 — Plots of vital signs for individual mice. Individual plots of pulse rate (PR), respiratory rate (fr) and arterial haemoglobin saturation (SpO2) for each mouse by after administration of medetomidine-ketamine (MK) or dexmedetomidine-ketamine (DK) by the intraperitoneal (IP) or subcutaneous (SC) route over time used for statistical analysis. The individual mouse identification number is located at the bottom left corner of each plot. [file 1746-6148-9-48-S2.pdf]
